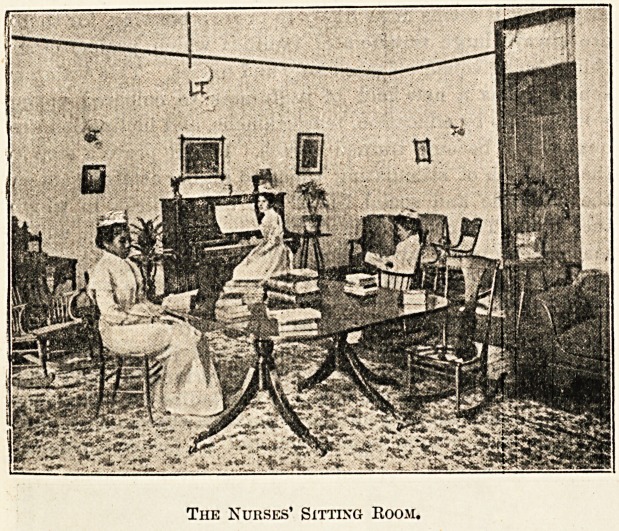# "The Hospital" Nursing Mirror

**Published:** 1901-06-08

**Authors:** 


### "The Hospital" Nursing Mirror.

Being the Nursing Section of "The Hospital."

[Contributions for this Section of "The Hospital" should be addressed to the Editor, "The Hospital" Nursing Mirror, 28 & 29 Southampton Street Strand, London, W.C.]

### Notes on News from the Nursing World.

### QUEEN ALEXANDRA AND "QUEEN'S NURSES."

This year the new nurses who have been added to the roll of Queen's Nurses will have the gratification of receiving their badges and certificates from Queen Alexandra. Last July the function was undertaken by Princess Henry of Battenberg, acting for Queen Victoria; but next month Queen Alexandra, who succeeds the late Sovereign as patroness, will herself personally make the distribution. The announcement, we are sure, will be received with the most intense satisfaction, not only by the nurses who are to be the recipients of the badges, but also by all who are attached to Queen Victoria's Jubilee Institute. The active interest thus manifested by the Queen, at the outset of the present reign, in the nursing movement, is yet another proof of the fact that her Majesty graciously intends to continue to afford it all the help and encouragement which it is in her power to bestow.

### HONOURS FOR AUSTRALIAN NURSES.

On Saturday, at Government House, Sydney, the Duke of Cornwall and York presented war medals to Nursing Sisters Martin and Woodward in recognition of their services in tending sick and wounded soldiers in South Africa. This is a sufficient answer to the statement that the medals are not to apply to Colonial nurses, and we hope to hear of further presentations.

### SCOTLAND AND THE WOMEN'S MEMORIAL TO QUEEN VICTORIA.

In response to the invitation of the Scottish Council of Queen Victoria's Jubilee Institute for Nurses, the Duchess of Buccleuch and Queensberry has agreed to become president, and to appoint a committee for a general appeal in Scotland for the further endowment of the Edinburgh centre as a memorial of the late Queen. But, owing to the fact that collections for the national memorial and various war funds are still before the public, it has been decided to postpone for a time any national organisation in the counties of Scotland. In the interval, private efforts will, however, be made to obtain contributions from persons interested in the scheme. If the interval is not unduly prolonged, this may prove to be a wise course.

### THE WAR OFFICE AND COLONIAL NURSES.

Not for the first time the complaint is made that the authorities of the War Office have treated Colonial nurses very unfairly. On this occasion a member of the Royal British Nurses' Association writes from South Africa to express her indignation "at the slurs and slights cast on Colonial nurses on account of their nationality and their supposed lack of nursing status just because some of them happened to be trained in South Africa." She declares that she herself was at first refused a post, though she has a certificate from one of the leading London training schools, and has held important hospital posts in South Africa; and she mentions another specific instance of the treatment which she condemns. "A Colonial nurse," she says, "who was appointed superintendent of a military hospital in South Africa, called at the War Office in London to report herself before returning to active duty in Natal. She was brusquely informed that 'the War Office did not recognise Colonial nurses as being in their employ. It was merely a local arrangement.'" There is every reason why nurses calling at the War Office should be treated with courtesy, but it is obviously impossible that the Army Medical Department can accept responsibility in respect to any who are not members of the Army Service, or the Army Service Reserve. The arguable question is whether the officials of the department in South Africa ought not at the outset of the war to have been instructed to admit to the Reserve a number of well-qualified Colonial nurses. This would have been a much better plan than that of employing such nurses as stop-gaps; and we fully recognise the force of the plea that their experience of the semi-tropical diseases of South Africa constituted a special argument in favour of utilising their services.

### THE WAR NURSES.

We are glad to learn from Johannesburg that Nursing Sister Richardson, who has suffered from a severe attack of enteric fever, is pronounced out of danger. There have also been discharged from hospital to duty Nursing Sisters Violet Isabel Lamb, Agnes Davidson, Amy Madeline Turner, and Frances Coralie Holcroft. Sister Culverwell's position is reported as grave, but improving; while that of Sister Webster was, on May 31st, critical. Last Friday Sisters L. G. Cooley, A. A. Flowers, J. S. Craw, and A. B. Conyngham arrived in the *Mohawk* on leave, and Sisters B. E. Caws, L. Schröder, H. M. Shaw, L. M. Tippets, and E. M. Denny have arrived in the *Bavaria*. Sister Harvest has been invalided home. Sisters T. Mason and S. M. Stanford are on board the *Tintagel*, which is expected at Southampton on Saturday week.

### THE NURSING OF COLOURED PATIENTS.

It appears that some of the Natal people are extremely disgusted that "European ladies" should be employed at Grey's Hospital, Maritzburg, to nurse Kaffirs and coolies. Their view, judging by the letter of a correspondent of the *Natal Witness*, is that it is degrading for "cultured ladies" to minister to the needs of coloured patients, and he asks, "why are not coloured nurses or orderlies provided for this work?" Happily, no objection has been raised by the European nurses themselves, who, we venture to think, are as willing to do their best to alleviate the sufferings of coloured, as of white, patients. In nursing, the racial distinctions which trouble cultured colonials are, or should be, unknown. If, however, they are felt as a serious difficulty, we would recommend to the notice of those who object to European ladies nursing coloured patients the example of the Americans who started the Training School for Coloured Nurses at New York, described on another page; clearly it will not do to leave these people to untrained nursing.

### ARCADES AMBO.

The Belfast Board of Guardians have sent a resolution to the Armagh Board of Guardians expressing their "appreciation of the action of the Armagh Board in initiating the proceedings which have resulted in the Lord Lieutenant disallowing the order of the Local Government Board of February 4th, 1901. "A fellow feeling makes us wondrous kind," but both the Armagh and the Belfast Guardians may find that the victory which was gained the other day may have been more disastrous than defeat. The Chairman of the Armagh Board does not, in fact, imagine that the Local Government Board mean to let matters rest. On the contrary, he anticipates that they will shortly make some regulations with regard to the nursing arrangements of the Armagh Workhouse Infirmary. The sooner and the more stringent the regulations the better. The authority of the Local Government Board would soon be undermined if they permitted either the Armagh Guardians, or the Armagh and the Belfast Guardians combined, to set them at defiance.

### ENGLISH BOARDS OF GUARDIANS AND THE NURSING QUESTION.

Some of the English Boards of Guardians seem ambitious to emulate the example of the Irish Boards in their treatment of the nursing question. As a comment on Mr. Bastow's speech at the meeting of the Bristol Board of Guardians, which was quoted in our last issue, a correspondent states that while he was vapouring about nurses walking about the streets, he must have been aware that until a few weeks ago two of the nurses in the employ of his Board had about 130 patients to attend to, and that for some time nurses at Barton Regis were not allowed out on pass, on account of the heavy duties devolving upon them. In the East of England, the resignation of the superintendent and another nurse at the Oulton Workhouse Infirmary has revealed the fact that constant friction between the master and the officials under him, in consequence of the regulations of the Board of Guardians, is the cause of frequent changes in the staff; while at Hayfield in Yorkshire the difficulty in keeping nurses provoked such an acrimonious discussion at a meeting of the guardians, that a scene of disorder occurred. A newly-appointed nurse who complained of the quality of the food given to her was informed that she would be permitted to procure her own rations in future. But there ought not to be any necessity for such an expedient.

### THE PREFERENCE OF RIPON.

It is highly significant that the Ripon people who were asked to support the establishment of a Nurses' Home and the laying-out of a recreation ground as a memorial of the golden wedding of Lord and Lady Ripon have contributed £2,135 towards the former, and £204 towards the latter. Recreation grounds are admirable in their way, but it is clear that in the Yorkshire city much more is thought of the needs of the sick and suffering than of the enjoyment of the healthy and happy. The preference is creditable, for the healthy and happy can take care of their own interests, and get plenty of enjoyment out of life, even without a recreation ground.

### A START AT HASTINGS.

It is rather astonishing that the people of Hastings have only just decided to form a District Nursing Association. Perhaps the explanation of their tardiness lies in the fact that until the end of last year, and for some time previously, a resident had, at her own expense, provided a nurse who worked in the old part of the town. In December, however, it got wind that the resident who supplied the nurse was leaving Hastings, and could not therefore continue the arrangement. Then, the prospect of losing the nurse created so much consternation that a small committee was appointed to see what could be done. The formation of the Association which was last month called into existence has been greatly expedited by the liberality of the Rev. W. C. Sayer-Milward, who offered to convey to trustees a suitable house for the purpose of a nurses' home. The promoters of the movement have very wisely counted the cost of the undertaking before embarking upon it, and their calculation is that £180 a year is needed for the first nurse, every additional one involving an expenditure of £65 to £70. This is a reasonable computation, and for the information of other towns, we may say that the £180 is made up as follows:—Cost of living, £78; coal, £10; servant's wages, £10; rates and taxes, £15; nurse's salary, about £40; sundries, £17. The influential attendance at the meeting over which Lord Brassey presided, and the enthusiasm of the speakers, justify the belief that the organisation will soon be placed on a firm footing. Dr. Shorter made a suggestion which may possibly be adopted. He proposed that instead of having a statue of the Queen as the local memorial to the late Sovereign, the townpeople should raise £1,000 for a nurses' institute. Even if the proposal has come too late, there is no doubt of the popularity of the nursing movement.

### A DECISION REVERSED AT RUTHIN.

We mentioned a week or two ago that the Ruthin Board of Guardians had declined to continue the grant of £5 per annum towards the Nursing Institution. Apparently, the determination was arrived at in haste, and has been repented of at leisure, for at the last meeting of the Board, Mr. H. Williams having moved, and Mr. Simon seconded, that the grant be made, thirteen voted for the motion and four against. Probably a letter from the medical officer of the workhouse, in which he expressed his hope that the Board had not finally resolved to dispense with the nurse's services, and pointing out that if they did so they would incur an expense double the amount contributed for private nursing, created an impression. At any rate, no one supported the protest of the guardian who strongly objected to the reopening of the question; and the Ruthin Board is free from the stigma of refusing to recognise the value of services which, in the words of the medical officer, "added immensely to the comfort of the paupers."

### A YEAR'S WORK AT CROOK.

The first annual meeting of a nursing association is always an interesting event. But it is not always so satisfactory as the first meeting at Crook in Durham. The report for the year showed that the number of visits paid were 1,620, and the number of patients treated 374; and that while the receipts amounted to £244 9s. 5d., the expenditure was only £184 5s. 10d., leaving a balance of £60 in hand. But even more encouraging than this substantial balance to the good was the fact that the workmen subscribed £101 5s. 11d., or not very far short of half the total contributed. We heartily congratulate the colliers of Crook on the handsome manner in which they have supported an organisation founded especially for their benefit. Their appreciation of the work of the nurses in their midst shows that the example of Messrs. Pease, the largest employers of labour in the district, who have done so much to promote the nursing movement, has been contagious.

### INSTRUCTION IN FIRST AID AND HOME NURSING.

Twelve months ago the London School Board appointed Dr. R. J. Collie to reorganise and superintend the ambulance and nursing classes of the continuation schools. He has just presented a report on the new scheme, which provides that the ambulance course shall consist of twelve meetings, six conducted by a qualified medical practitioner, and six by one of the Board's teachers, who is to assist the doctor, and on each alternate evening, in the doctor's absence, to conduct the class. The nursing classes may be taught by a certificated hospital nurse, or by one of the Board's teachers who holds the Board's special teacher's certificate in home nursing, or by a doctor. A few classes held in the poorer districts have been entrusted to hospital-trained nurses, selected by the Evening Schools Committee. These classes, it appears from the report, have been attended, well instructed, and, in the opinion of Dr. Collie, are bound to be productive of much good. The lecturers and teachers, he says, have been instructed to make it their object to show that the common and inexpensive things of everyday life may be utilised in emergencies: that, though hospital treatment and hospital-trained nurses are admittedly the best for most cases of serious illness amongst the poor, there is always a considerable number of the sick who are either inadmissible to hospitals or who for various reasons prefer to be nursed in their own homes by their relatives and friends, and that the instruction given at the home for nursing is intended to assist the pupils to be of service at such times. It is satisfactory to hear from Dr. Collie that the pupils who attend the classes "take an intelligent interest in and readily master the details of teaching." It is to be hoped, however, that they will not receive any form of certificate which will tempt these little people to strut about and call themselves "nurses." In the last term there were 191 ambulance and nursing classes; this year they have increased to 460, of which 300 have been on first aid to the injured and 160 on home nursing. There are thirteen trained nurses on the staff.

### A NURSES' HOME FOR CHRISTCHURCH.

The Christchurch Board of Guardians have adopted the recommendations of the building committee with respect to the nurses' home. The only fault found by the Local Government Board with the plans was the proximity of the new cesspool which is to receive the drainage to the buildings, and this objection is to be met. In inviting tenders for the erection of the home, the Guardians stipulate that it must be completed in six months from the date of the commencement of the work. It may therefore be assumed that the nurses of the Workhouse Infirmary will enter into possession of their new quarters before Christmas.

### NURSES AT THE MEDICAL EXHIBITION.

A large number of nurses visited the Medical, Surgical, and Hygienic Exhibition, at Queen's Hall, Langham Place, last week; indeed, they were there in such crowds that when our representative inquired as to numbers the reply was that it had been found useless to try and keep any record of names in the visitors' book. Perhaps the majority gravitated to the stalls on which invalid foods were shown; for it was a good opportunity to pick up useful hints for varying the diet of patients. District bags and nursing requisites attracted a good many. A small group were discussing the advantages of treadle-taps for fixed basins, which left the hands free during the progress of an operation. An ever changing crowd hovered round the luminous electric radiators shown by the Dowsing Company, which the nurse in charge of the electrical department was explaining. The radiant heat and light bath, as used on the hospital ship *Maine* for the treatment of rheumatism and sciatica, attracted much attention, and so did the lupus cure, although it was not at work owing to the enormously strong current required. A small electric kettle which was put on to boil without any visible heat must have reminded South African nurses of the voyages home with invalids. There was a very attractive programme of orchestral music each afternoon and evening while the exhibition was open; this was an additional pleasure to many hardworking members of the profession who rarely have time to go to a concert, and the gallery with its comfortable seats was a pleasant place to rest in when one had gone the round of the stalls.

### CORNISH GUARDIANS AND DISTRICT NURSING.

At the fortnightly meeting of the Redruth Board of Guardians application was made by Mrs. Powys Rogers, president of Gwennap Nursing Association, for a subscription towards the nursing funds. The Gwennap Guardians supported the request, and the Guardians of other parishes thought that if Gwennap received a grant other nursing associations in the Union should be treated on the same lines. On the whole, the application, which was postponed until the next meeting for further inquiries, was favourably entertained, one guardian proposing a subscription of £2 2s., another seconding with the proviso that other nursing associations in the Union be similarly treated. The poor in the Redruth Union are certainly being well attended by district nurses. Camborne, Tuckingmill, and Troon have three nurses, sometimes more, constantly employed, Gwennap one, and Redruth one, and their timely help in cases of sickness must do much towards enabling the poor to remain in their own homes rather than throw themselves on the rates. The further action of the guardians will be watched with interest by residents of these important centres of the mining population of Cornwall.

### THE QUEEN'S LEGACY.

A Queen's nurse, when visiting an old man in South Wales, was asked by him "what was that thing she had on her arm?" On being told that it was the badge worn by the Queen's nurses, he wanted to know "if the Queen had left it her when she died."

### SHORT ITEMS.

It was decided at a meeting presided over by the Mayor of Peterborough last week to raise local funds for the Queen Victoria Nurses' Endowment Fund, as a local memorial to the late Queen.—Miss Rachel Titherington, who a few weeks ago resigned the post of matron at the Ealing Hospital, which she had filled to the great satisfaction of the committee and all who came into contact with her, has started a private nursing home at Ealing.—The village of Hedgend, near Botley, in Hampshire, has lately, through the kindness of the owner of Botley Grange, obtained the services of a district nurse. Mr. Foord has built and furnished a very comfortable little cottage in the midst of the village, quite near the church. Nurse Gammon, who has had previous experience in district nursing, will be a great help to the villagers and to the medical man who lives some miles away.

### Lectures to Nurses on Medicine and Medical Nursing.

By NORMAN DALTON, M.D. London, F.R.C.P., Physician to King's College Hospital.

### LECTURE XII.—THE NURSING OF CASES OF MITRAL DISEASE.

THE condition which exists in a bad case of mitral disease is most distressing to witness. The constant shortness of breath, the impossibility of lying down, and the terrible desire for sleep may last for days and weeks and, even if occasional improvement occur, for months, and the resources of the nurse are tried to the utmost. She must remember that, apart from the action of drugs, the patient's chance of recovery depends on his muscular strength. As long as the muscle of the right ventricle is strong enough to empty its cavity fairly well at each contraction and also to keep the blood going through the lungs in spite of the congestion there, life will be maintained, and, at any moment, the action of the drugs or the recuperative power gained by rest may succeed in re-establishing compensation, upon which the bad symptoms will subside. Again, as long as the voluntary muscles of respiration remain strong enough to work hard, there is a chance that enough oxygen may be supplied to the blood to support life until the heart begins to mend. Therefore the life of the patient depends on his strength and endurance, and he must be treated like an athlete engaged in some severe competition.

It may be argued against this statement that we sometimes see a weak woman "pull through" a severe illness of this kind, while a strong man may die in a few days. But comparatively feeble women may possess a great power of endurance. The man feels the sense of shortness of breath more acutely than the woman, and uses his strength to its utmost to relieve it, and so may soon become exhausted; whereas a woman can suffer more, and so husbands her strength that she can keep up the fight longer. It is well known that fat people of either sex bear the strain badly, and soon succumb.

It follows from what I have said that all the patient's strength must be reserved for pumping the air into his lungs, so that on no account must he be allowed to do anything for himself. The nurse must, as far as possible, divine his wants and forestall them. If he wishes to change his position he must be lifted, and in particular sudden movements, especially the act of rising from a lying to a sitting position, must be prevented, because this change throws a great strain upon the heart. Talking, also, exhausts the strength, and a good nurse should be clever in interpreting signs.

It is important to make the patient as comfortable as possible in the sitting position, which he is obliged to assume in order to breathe. A bed-rest is useful as a support to the back, and if a regular one cannot be procured, a substitute can usually be made out of a box or some boards, but it is essential that the rest should be firmly fixed, so that the patient has no sense of insecurity at his back. The rest should be arranged so as to press firmly against the sacrum and lumbar region and lightly against the dorsal region where the lungs are. It is usually covered with pillows. The position of the patient as regards the bed-rest requires to be carefully watched. As he gets weaker he tends to slip downwards. This brings the sacrum on to the bed, while the dorsal region presses strongly on the bed-rest; the shoulders are arched forward, and the chin presses on the chest, which is a very bad position to breathe in. By careful observation and the re-adjustment of the supports a nurse may prevent the adoption of this attitude, and, if it should occur, she must correct it at once. Great discretion is necessary in this matter, because it is obviously not wise to be always pulling at the pillows. Another great help to the patient is to have some kind of rest in front of him on which he can place his elbows when he leans forward, and so get a good purchase for his extraordinary muscles of respiration. Bed-tables are made for this purpose, but something of the kind can often be improvised, only it must be very strong and firm. With all these helps the patient's attitude becomes most irksome. The hips and buttocks ache, and the legs being stretched out straight get stiff and cramped. Friction of the legs and hips may give relief. Many cases, however, are more comfortable in a strong, straight-backed armchair; and, if the patient be not too heavy to lift, a change from the bed to the chair may increase his comfort. Of course, this change must never be made without the doctor's orders. Bed-sores must be guarded against, and it may be necessary to use a water-cushion or water-bed, though the latter is not a good thing from the point of view of the breathing.

If the patient be allowed to sit up, the dropsy in the legs may get worse. This is not an evil, because if the dropsy be unavoidable (on account of the state of the heart), it is better that it should be in the legs than in the lungs; particularly as, if necessary, the fluid can be withdrawn from the legs by tapping. It follows that in cardiac dropsy no good is obtained by keeping the legs up or by raising the foot of the bed. On the other hand, in dropsy due to the blocking of veins, such as occurs after typhoid fever, or after a confinement, the legs must never be allowed to hang down. Every precaution must be taken to guard the legs from scratches, and if they occur they must be covered by antiseptic lotions at once.

As the maintenance of the strength is so important, the patient must, if asleep, be waked to take his nourishment and medicine at the appointed hours. If the patient be able to sleep for any length of time he is either really better or he has given up the fight and his sleep is the coma which precedes death. If he be really better, he will fall asleep again as soon as he has swallowed his food, so that there need be no hesitation in disturbing him. If he be giving up the struggle, his last chance consists in rousing him to take his food, medicine, or stimulant. The food should be given in small quantity at a time, because a distended stomach presses the diaphragm upwards and interferes with its full action. As the quantity must be small, nourishment must be given frequently.

The mind and the will exercise a great influence over the muscular strength, consequently the nurse must encourage her patient in every way. The effect of a few cheering words is very stimulating. In some cases patients suffer after a few days from great pain round the lower ribs. Apart from lung complications, this is usually due to the exhaustion of the diaphragm and respiratory muscles brought on by overaction and by coughing. Various applications are ordered for this pain, but friction by the nurse of the chest all round may be tried at any time. More will be said about the nursing of mitral disease in the next lecture.

### To Nurses.

**WE invite contributions from any of our readers, and shall be glad to pay for "Notes on News from the Nursing World," or for articles describing nursing experiences, or dealing with any nursing question from an original point of view. The minimum payment for contributions is 5s., but we welcome interesting contributions of a column, or a page, in length. It may be added that notices of entertainments, presentations, and deaths are not paid for, but, of course, we are always glad to receive them. All rejected manuscripts are returned in due course, and all payments for manuscripts used are made as early as possible after the beginning of each quarter.**

### A Day in my District.

By a Queen's Nurse in the Country.

7 a.m. There is a knock at my door followed by the entrance of the little maid where I lodge. She accosts me with a pleasant "Good morning," puts down my can of hot water, and gives me a nice cup of hot tea. I thank her and inquire as to the state of the weather. I am pleased to learn that the morning is fine. Having enjoyed my tea, I rise, dress, open windows top and bottom, stripping my bed to the morning air. Breakfast is ready at eight. After breakfast I make my bed, tidy my room, read for a little while, and prepare to go out at nine. The district is quite in the country, very scattered, the villages being considerably apart.

### The First Visit.

My first visit is to a child living at a distance of two miles from my lodgings. The season of the year is spring; the morning is lovely, balmy air and bright sunshine everywhere. I take the shortest way across a number of fields—a route not to be chosen in wet weather. As I walk I admire anew the beauty of the scene surrounding me. Nature is rejoicing on every hand. The fields rich in green, through which various spring flowers appear and shed their fragrance, afford a soft and pleasant footpath. A river winds its quiet way on my left hand. Trees grow on its banks; ferns abound in the shade, their growth increased and beauty enhanced by the moisture of the soil and the shade afforded by the trees growing in their vicinity. Birds are singing on many boughs, while above my head larks vie with each other in their morning songs. To the right is a thick wood, the home of many squirrels—it now resounds with the note of the cuckoo. Rabbits disappear in the underwood at the sound of my approach. These are seen in greater numbers in the evening hours; I frequently disturb them at their evening meal. Wending my way, I leave river and wood behind, follow a path through a field now green with early wheat. At the head of this field there is a quarry, enclosed by ivy-grown walls. I look over and covet the ferns growing lower down on its walls and out of my reach. Two goats are tethered in the waste ground close by; they are tugging at their chains, vainly struggling for their emancipation. A few more steps, and the house to which I am bound emerges close to hand. My patient is a small boy of seven, convalescent from croupous pneumonia. I take his temperature, pulse, and respiration, write out and leave report for the doctor. The mother of the child supplies hot water, soap, flannel, and towels. I remove the sheets from the bed, wrap the patient in a blanket, taking off night-shirt and wool jacket (the latter I made and gave at an earlier date). I put sheets, shirt and jacket to air, and proceed to wash over my patient. Having dried and dressed him, I make the bed, the mother assisting me.

### Two Ordinary Patients.

My next patient lives close to my lodgings, so I retrace my steps. This patient had been a laundress; she suffers from ulceration of both legs. She is a pleasant old woman, very chatty, but very deaf. She tells me many tales of the neighbourhood in which she has all her life resided, while I am doing the dressing. No. 3 is an old man, bed-ridden for many months. He lives in the next village, distant about a mile and a half. Like the old woman he, too, delights in recalling old times. I am sure, as he lies month after month in his quiet little room, it gives him pleasure to live over again these scenes of his life which were happier than the present are. To-day he is as usual: there is no disease from which he suffers; his is a general and gradual decay. His niece lives with him; she tells me at times how much he prizes my coming, although at the first he partly resented the needful washing. I now wash him, attend to his back, make and change his bed, his niece assisting with the bed-making. Putting a few touches to the arrangement of the room, and seeking to impress the necessity of ventilation on this niece—oh, so hard to teach—I prepare to leave the old man and return to my lodgings.

### An Urgent Case.

It is dinner time when I get back. Ere that meal is over a message is given me from the doctor requesting me to go to a child, suffering from acute laryngitis. The child's home is at the other end of the village from which I have just come. The case requires immediate attention, so I give a steam kettle to messenger, who is returning, and hurry after him. Upon arrival I find the child suffering acutely and the mother very distressed. I ask for and obtain two large clothes-horses for the purpose of erecting a steam tent. With these we surround the bed, leaving an opening on the side nearest the fire. We pin on our hangings and cover over the roof. I now make the child more comfortable, renewing the fomentation applied to the throat, at the same time showing the mother a better way of fomenting. I hang up a thermometer inside the tent, put the steaming kettle in position, also arranging that there shall be a supply of fresh air without a draught being caused.

Ere I depart the mother shows me another delicate child. I try to allay her anxiety, advising her to let her children have all the sunshine she can.

### Another Summons.

I get back to tea and find awaiting me a summons to attend a confinement. I hastily drink a cup of tea, not stopping to eat bread, I take up my midwifery bag and start for the house of my patient, distant three miles. The message was sent shortly after I went out to the child, consequently I feel anxious lest I should not be in time. I do not wait to admire the beauty through which I pass—my thoughts are with my patient. Soon the ground is traversed. I run quickly up my patient's garden, knock at the open door, and scarcely waiting for an answer, enter. What do I find? my patient at tea with her husband and a friend. I inquire if I had been sent for, thinking there was some mistake. I am assured to the contrary, so I quietly await events. The husband goes out in a few minutes, and I proceed to make inquiries. I ask about the pains, and am told that they are weak and at long intervals. I obtain some hot water; the patient takes me to her bedroom. I prepare my lotion, wash and disinfect my hand. During the first pain I examine. I find the os dilated a little more than the size of a shilling, membranes intact and a cranial presentation.

### Long Hours of Waiting.

On making this discovery I felt very much disappointed. I think of my tea that I might have taken in less hurry; I regret my haste in walking, and dare not look forward to the long hours of waiting before me. I tell the patient she has brought me much too soon; she excuses herself by pleading the distance to my lodgings, telling me at the same time how she suffered on a previous occasion by not sending soon enough. I prepare a simple enema which I give, meanwhile considering what I shall do. I decide to remain with the woman—to go back would necessitate a return in a few hours, involving a long walk both ways in addition night would shortly fall, adding loneliness to walking. I advise the patient to keep about and I go out and explore the garden. I return to my patient rested and cheered. There is a nice fire in her bedroom; together we prepare all things necessary. She is very cheerful and chats pleasantly between her pains, which are now becoming more frequent. About midnight her friend gives me some tea and bread and butter which I gratefully accept. At one o'clock I examine patient; finding the os almost dilated, I rupture the membranes and keep the patient in bed. At 2.30 the baby is born. There is a little delay before the placenta is expelled; there are no complications.

Home at Five a.m.

I put patient comfortable, wash the baby, and tidy the room, the friend kindly assisting. Taking one more look at the patient and counting her pulse I prepare for home. The husband walks part of the way with me, but, day breaking, I send him back. It is five when I reach my lodgings; my supper is still on the table; I take some and go to bed. This is what one finds sometimes in country nursing. Of course, there are many days in which there is little work; many nights, too, in which the nurse may sleep the round of the clock if so disposed, and a bicycle—which since writing this I have learned to ride—is a great economiser of time and exertion.

### Nursing in a Seminary in South Africa.

By a Correspondent in Cape Colony.

When I first came out to Capetown I was not very strong and did not care to take up private nursing as a permanent thing, so I thought I should look out for something less trying, and through the kindness of the Lady Superintendent of a nursing institute here I secured a post as resident nurse in the Huguenot College and Seminary in Wellington, Cape Colony. I went there thinking to stay three, or perhaps six, months, but I was so happy and grew so attached to the place and people that I remained for sixteen months. In the college and seminary we had upwards of 170 boarders, 32 teachers, three lady principals, and two or three secretaries. Being a good way from Capetown a resident nurse was essential; there were two or three doctors in the village but what was really required was someone who knew when a doctor was needed and when it was not necessary to fetch him. Besides, there were many emergencies, when hard-worked teachers, housekeepers and principals had no time to see after sick people, and they would have fared badly had not a nurse been on the spot to see to their comfort. I arrived at the seminary, where I took up my abode in June, the winter season.

Absence of Friction.

I was very kindly welcomed by the Lady Principal, and introduced to several of the teachers; all were most kind and genial, and I may say here that I never once felt lonely or strange in any way during all those pleasant months I stayed in Wellington. I made many dear friends amongst the teachers and found them highly intellectual women, gathered from different quarters of the globe. I believe the cosmopolitan nature of the place struck me almost immediately, and later the absence of friction in such a mixture of nationalities amazed me. Our girls for the most part were Dutch, many of them just fresh from up-country farmhouses; in character kindly, good-natured, easy going, and somewhat lazy and indolent. There were, however, many exceptions to the rule of the last-named characteristic, often there was ambition, firmness of purpose, great staying and enduring powers in the Dutch girl, and I grew very fond of any I really knew well. I did not confine my attention only to the sick ones, but tried to grow acquainted with as many as it was possible in such a big family.

The Girl Patients.

I found the girls very good patients; obedient and patient in any suffering. It was amusing to note the difference in the girls; some would want to lie in bed for the most trifling ailment, groaning and moaning over imaginary aches and pains, whilst others would go on enduring real suffering rather than be put to bed and miss the schooling which they counted so precious. I had many funny experiences with my girls. How often have I been sent for to see a patient and found instead a poor little lonely homesick girl, who wanted no doctoring, but merely a kind word and a wee bit of mothering.

The Buildings.

The college and seminary buildings stand in their own grounds; a long line of houses form the seminary, teachers' and girls' bedrooms, schoolrooms, music rooms, &c.; opposite these buildings are the little kindergarten, and further on is the hall for class rooms and all big gatherings, social or otherwise. Devotions are held in the large room every morning, and service, morning, afternoon, and evening, every Sunday. At the top of the grounds is the college—a very handsome building, and beautifully furnished and fitted out. It would take too long to describe the whole place, and might not prove interesting to those far away.

Outside Duties.

Besides my duties as nurse, I had others which cannot be classed in that province. I was in charge of the seminary linen, and saw to the sorting, distributing, marking, &c. I went a round of the girls' bedrooms twice a week in each house to see that all were kept in order. I acted as sanitary inspectress, going a round of the grounds, reporting any disagreeable odours, imperfections in drainage, &c., and I also undertook some of the teachers' duties, such as going the last round once a week to see that all lights were out and no midnight revels taking place. Altogether I was kept very busy, and always had someone sick on my hands.

The Cases.

During my stay I had cases of typhoid fever, measles, mumps, chicken-pox, tonsillitis, peritonitis, laryngitis, fractured humerus, severe scald, influenza, bronchitis, pneumonia, and pleurisy. I also gave simple nursing lessons to mission students who were preparing for work in the foreign fields. I taught them just what I thought would be most useful to them. Poultice-making and bandaging, how to attend the sick, changing sheets of bed-ridden patients, to take temperature, to dress wounds, and a little sick cookery. Wellington is a very pretty little spot; the most beautiful sunsets are to be seen there, when the mountains, which form almost a complete circle round the little village, are flooded with a glorious pink, purple, or orange. A very nice spirit of goodfellowship and kindness pervades the college and seminary; even during the strife and misery of war, wonderful forbearance and kindliness prevailed. All are working together, teachers and girls; there is little real unpleasantness and few cases of misbehaviour. As far as it is possible, every individual character is studied and allowances made, and education is not the only thing thought of, but a girl in the Huguenot Seminary and College has every chance of forming a fine and noble character, for there are many ready to give sympathy and help to all who ask for it, and the keynote of the place is, faithful service to God and a working out of practical Christianity.

### Nursing in the Coloured Home and Hospital, New York.

By an American Correspondent.

In 1839, seven ladies met at the house of Mrs. Banyer, a daughter of the Hon. John Jay, "to take into consideration the destitute condition of the coloured population in New York, and to devise a plan for the alleviation of their unobtrusive sufferings." The initial meeting for the relief of the coloured people of the city was fitly associated with the

THE COLOURED HOME AND HOSPITAL, NEW YORK.

family of Jay. The name is not only justly honoured owing to the distinguished services of the elder John Jay, but it was rendered conspicuous in the great anti-slavery movement by the writings of Judge William Jay, and still later by the advocacy of its doctrines by the then John Jay, jun. At a subsequent meeting an organisation was perfected with the title of "The Society for the Relief of Worthy Aged Coloured Persons." Miss Jay contributed $1,000, to be especially appropriated "to the relief of destitute sick coloured men and women." The growth of the society was necessarily slow, for it was not regarded with the same popular favour as other similar institutions devoted to the care of the sick-poor of the white population. But it received several grants from the Legislature and donations from private individuals who sympathised with this form of charity. Thus, it was enabled to continue and enlarge its work, until from twelve persons who were presented at the first meeting as worthy of its support, upwards of 800 annually passed through its wards. The society was incorporated by the Legislature in 1845 under the title of "The Society for the Support of the Coloured Home." It was first located on the west side of New York, but afterwards occupied a building on 42nd Street. In 1848 the society purchased forty lots of ground on First Avenue between 64 and 65 Streets, on which it erected buildings, which were completed in 1849. The home consisted of four departments, viz., the home proper for the aged and indigent persons, the hospital for the sick, the lying-in department, and the nursery. The average number of inmates was 215. The inmates of the home department were feeble old men and women, some of whom had been slaves in this State and New Jersey. The patients in the hospital belonged to the indigent class of the city, and had to be admitted through the Department of Charities, which was responsible for their care, and allowed the home a small *per capita* sum for their maintenance and treatment. In 1898 it was decided to sell this property and with the proceeds to purchase a new site and build and equip a new home and hospital. Steps were accordingly taken to sell the First Avenue lots, which during the fifty years of possession had largely increased in value. A site was selected in the suburban district admirably adapted for the institution and a series of hospital buildings have been erected thereon which are not surpassed in this State, in all modern sanitary arrangements and scientific equipments.

The Training School.

But the lady managers were not content simply to minister to the physical wants of the aged and infirm, the sick poor, and the orphans of the coloured people of the city; they wisely determined to utilise the advantages of the institution for the education of competent young coloured women to become trained nurses. This is among the first efforts to educate coloured women for the profession of scientific nursing. There are schools of the kind in this country, most of which are located in the hospitals of the Southern States. It is a matter of surprise that the movement to train coloured nurses was so long delayed considering their peculiar aptitude for domestic service and their skilfulness in performing the duties of nurse when the opportunity offers. It has been alleged that their colour would prove a fatal obstacle to success in general practice in every community. But it seems more likely to prove a blessing, for there are always large numbers of families which prefer, other things being equal, coloured help and nurses. This training school opens a new field of usefulness to coloured young women, who by education, temperament, and inclination are qualified to become trained nurses. It will prove a step in the direction of stimulating them to higher attainments in the domestic and social life of the communities in which they live. The course of instruction is very complete, and in many respects the advantages of the hospital for the training of nurses excel those offered by some of the older and larger institutions.

Sixty Years Ago and Now.

The actual instruction of the nurses in the school is exceptionally thorough in both the principles and the practice of the art of nursing. If the ladies who sixty-one years ago met "to take into consideration the destitute condition of our coloured population, and to devise a plan for

THE NURSES.

the alleviation of their unobtrusive suffering," and consequently organised "The Society for the Relief of Worthy Aged Coloured Persons," could visit the magnificent institution beyond the Harlem dedicated to that service, their surprise and gratification at the fruition of their prayers and labours would be great. In one department they could see the "worthy aged coloured persons" neatly clothed, sitting in clean and nicely-furnished dormitories; in another the sick, whose "unobtrusive sufferings" so kindly appealed to their sympathies, now lying in beds which they would have regarded as luxurious; in a third, the poor emaciated consumptive, surrounded with every appliance for his comfort and possible recovery; in a fourth, the young mother with her child in a separate cottage, the furniture and conveniences of which are as dainty as were once furnished to the wealthiest; and, finally, the training school, preparing capable young women for the profession of scientific nursing.

### THE NURSES.

The Coloured Home and Hospital Training School for Nurses offers a two years' course of instruction to coloured women desirous of becoming professional nurses. The most acceptable age for candidates is from 21 to 35 years. With the application is sent a letter from a clergyman testifying to their good moral character and one from a physician certifying to sound health and unimpaired faculties. They are received at any time in the year when there is a vacancy. During the term of trial—two months—and previous to their entering the school, they are given an examination in reading, spelling, penmanship, and simple arithmetic. In the probationary term the pupils are boarded and lodged at the expense of the school, but receive no other payment. Those who prove satisfactory are accepted as pupil nurses. They reside in the institution and are expected

THE NURSES' SITTING ROOM.

to perform any duty assigned them by the superintendent, either to act as nurse in the hospital or be sent to private cases among the rich or poor. At the end of the first six months the nurses have to pass an examination and are required to sign an agreement to remain two years and to obey all rules of the institution. They are given a written and practical examination at the end of their junior year by the superintendent of the training school. The final examinations are conducted by the visiting physicians and surgeons. There is a written and also a practical examination consisting of nursing helpless patients, making and applying poultices, bandaging, cupping, &c. There are lectures and clinics by physicians and surgeons during nine months of the year, and side instruction and demonstration from time to time.

### HOURS OF DUTY AND PAY.

The nurses when on duty wear the dress prescribed by the Institution. The day nurses are on duty from 7 A.M. to 7 P.M., with an hour off duty for meals and an additional time for rest and exercise. They have one afternoon off each week, also part of Sunday. A vacation of two weeks each year is allowed. The pay for the first year is $6 a month, and $7 for the second year. This sum is allowed for dress, text-books, and other personal expenses of the nurses. It is not intended as salary, for the education given is considered an equivalent for services rendered. Board, lodging, and washing are furnished without charge.

### Nurses' Home at Richmond Infirmary.

It has long been felt that better accommodation for the nurses at Richmond Workhouse Infirmary was wanted; they are at present inadequately housed, and the night nurses are obliged for the sake of quiet to sleep in lodgings outside. This, however, will be altogether obviated when the new buildings, of which the foundation-stone was laid last week by the Mayor, Alderman Sir J. W. Szlumper, are finished. The work will probably take rather more than a year, for very extensive alterations are being carried out at a cost of £40,000; and six main blocks, with provision for 172 patients, and space for the open-air treatment of phthisis are being added.

The nurses' home is to be placed between the new lying-in ward and the male infirmary: it will consist of three floors, from each of which access will be obtained by short branches to the main corridor. The general arrangement on each floor is to be a central passage with rooms opening from it, and the architect has provided a secondary means of exit in case of fire by placing the indoor staircase at the opposite end to the outside corridor. Each passage is to be supplied with a fire hydrant. On the ground floor are to be the nurses' dining and sitting rooms, with separate dining-room for the subordinate nurses or wardmaids; and a private sitting-room with bedroom attached is to be reserved for the superintendent nurse. The kitchen is to be next to the dining-room, with a hatch between. Larder, linen-room, boot-room, and lavatories will be well placed, the last being at the end of the block; and it is intended to supply a nest of lockers, so that each nurse may have her own. The passage will be paved with terrazzo and warmed by two radiators. On each floor above will be 12 separate bedrooms, the top floor having four two-bedded rooms, so that 16 wardmaids may be housed. Below the ground level will be a box-room and a coal-cellar, and as the ground slopes slightly, light will be obtained without much difficulty. Mr. E. J. Partridge is the architect, Messrs. Soole are the builders, and Mr. A. G. Barley is acting as clerk of the works.

The stone-laying was a brilliant ceremony, and among others on the platform were the infirmary nurses in uniform. Mr. F. W. Dimbledy, Chairman of the Board of Guardians, said it was not a matter for surprise that additional buildings were required. The erection of the old workhouse for Richmond and Kew was undertaken by his Majesty George the Fourth, 116 years ago. Six parishes were now in the union, with a population of over 50,000. In the old days the system was for one old cronie to nurse another; there were no skilled nurses and very little supervision. Now they had to employ a good number of skilled nurses, and he believed it was true economy. The new nurses' home was being erected in accordance with modern requirements. They had had a little difficulty in the past. Very naturally, skilled nurses were not satisfied with the accommodation offered in old buildings, and so they had not always been able to retain the services of their nurses. They would, however, be very comfortable in their new home, where, he hoped, they would lead a happier life, with better facilities for the performance of their very important duties. The Local Government Board were very particular with regard to accommodation for the nurses. When the plans were submitted they wrote back to say that accommodation was required for the nurses' bicycles, and this had accordingly been provided for.

The new buildings will have a very charming outlook, for they are close to Richmond Park, where the fallow deer are tame enough to come up to the boundary wall for food; and the only drawback to the alterations is that they will do away entirely with the garden.

### Home from the War: a Chat with an Army Reserve Sister.

BY A CORRESPONDENT.

"I WAS very much struck," said Nursing Sister Denny in a chat I had with her the other day, "with the careful way the Netley orderlies removed the stretcher-cases from the ship. Of course you can't expect them always to be as careful as a nurse would be, but they did their best to avoid unnecessary suffering. The sergeant in charge was most attentive, and took every pains to make the removal as easy as possible."

"These were serious cases?"

"Yes; we brought eight or ten very serious ones home on the s.s. *Bavarian.* One was so ill, with a relapse after enteric, that I am afraid he will have been a good deal upset by the necessity of moving him. It was a case that in hospital we should never dream of moving, but, of course, he had to go to Netley."

"When did the landing take place?"

"On Saturday morning, about ten o'clock. We came into dock on Friday, and the Warwick Regiment went on shore that day; the sisters stayed to see that the sick were ready, and to make them as comfortable as possible for the journey."

"Did any of the sisters suffer from the voyage?"

"One was ill every time she went below; the rest of us were very much upset for two or three days, but we got over it. The heat and noise are very trying, and you are more conscious of the rolling movement of the vessel down in the hospital. The vessel rolled terribly after leaving the Cape, for we had only the troops on board, and did not take in our cargo till later."

### COMING HOME.

"You are home on leave, are you not?"

"Yes; I was at Kroonstadt, and had gone down to Wynberg for a short rest. I was not at the hospital then, but at Springfield, where there is a home for sick sisters or those for whom there is not enough accommodation in the sisters' quarters. They are not supposed to be roughing it, and so are not on field rations. The superintendent asked me whether I would like a passage home on a transport, and the offer of a visit to England after fourteen months away was too attractive to be refused, so I said I should. I was to be ready by 9 o'clock next morning in case I was sent for. Well, I got everything packed, and 9 o'clock came. The nurse who was sharing with me went off, and I thought I was going to be left, so I settled down to needlework. However, about 12 o'clock a cab came, and I had to put my things together and go off at once; I was on board, and away from land, almost before I knew what had happened!"

"The change must have been delightful."

"It was. But I have very much enjoyed being in South Africa; living under canvas is very healthy, except in the rainy season. The rain is not like English rain; it comes down as if it meant it, and it leaves a mark. The felt outside the tents was like clay; and some of the sisters used to go about in wading boots.

### NURSING DYSENTERY CASES AT KROONSTADT.

"We had very hard work at Kroonstadt with so many dysentery cases; some required even more care and watching than enterics, and it was pitiable to see the volunteers—many of them mothers' only sons, with no experience of roughing it—just as ill as they could be. They kept pouring in in such numbers. There was a good deal of trouble at one time about their brandy; one couldn't carry the bottles across to the sisters' quarters every time, and there was no place to lock them up in the tent; even when one of the sisters took a bottle across and hid it among clean sheets it disappeared: an orderly watched her and stole it. This is the only trouble I have had with orderlies, and these were not Englishmen; but it was a serious matter, for one man was so tipsy that he fell down behind a bed while carrying the lantern for me when I made my round at night."

### WASHING PATIENTS IN DIRTY WATER.

"Did you find it easy to work with the orderlies?"

"Yes, fairly so; but I should like to have done more for the patients than an army sister has time or opportunity for; for instance, the sisters are not supposed to make the beds, and anyone who has been ill knows what an infliction even the tiniest crumb is if it is left in the bed; it is a little matter, but quite capable of producing sores on a tender skin. The men so thoroughly appreciate all the attentions one can give them. Here is an instance of two different ways of doing a thing: I found an orderly going round washing the men without changing the water; one of them suggested that clean water would be nicer, and that he would be ready in a few minutes to take his turn. This was the reply: 'If you don't wash now you shan't wash at all, and if you won't use this water you shan't have any.' And this to a sick man! Of course he was upset. But these instances, as I said, were exceptions, and as a rule I found the orderlies most anxious to do their duty."

### TREK-OX AND JAM.

"When I arrived," continued Sister Denny, "the sisters thought they were very well off for food. We lived chiefly on trek-ox—and as he works so hard he is not very tender—and eternal jam; we had a great run of gooseberry jam, and then another few weeks of apricot; and one does get tired of it after a time. Of course, there were other things, and the tinned foods were excellent. We managed to get a few eggs from the village at about 6d. each, and tomatoes were 7d. or 8d. each. Butter was tinned, and the supply of fresh milk was limited; it was usually just enough to go round the hospital, and the sisters used condensed milk."

"Were you at Kroonstadt all the time?"

### AN ODD EXPERIENCE.

"No; I went out with Number Eleven to Wynberg; then I was transferred to Number Six, and I was very fortunate in being sent up-country to Johannesburg and Kimberley, and I had a few days at Bloemfontein, though I was not nursing there. At Johannesburg we had a very odd experience; the sisters lived in three private houses, next to each other, belonging to very rich Dutch people who had left them in a hurry, everything, even photographs, being just in their usual places as if the owners were there. An old Kaffir woman was in charge of one house, and she very highly disapproved of our presence. But we were most careful; we put all the valuables into one room and locked the door, and we took great care of the furniture. I felt so sorry for the people when we moved to another house, for we took everything with us—carpets and beds, &c. I knew how I should feel if my things were being carried off like that. But it is allowed in war, and I suppose compensation will be made. They were beautiful houses, magnificently furnished."

Sister Denny was much impressed with the barrenness of the veldt. "The faces of a detachment of Baden-Powell's police," she said, "made my heart ache. We met them in an open truck, going up, as we came down to Capetown. They looked so sick and tired, and I believe if we had said 'Come home to England with us,' they would have turned round and come. We knew the country they were going to."

Sister Denny was wearing the active service ribbon given to the nurses on board; it is an earnest of the medal that is to come.

### Everybody's Opinion.

[Correspondence on all subjects is invited, but we cannot in any way be responsible for the opinions expressed by our correspondents. No communication can be entertained if the name and address of the correspondent is not given, as a guarantee of good faith but not necessarily for publication, or unless one side of the paper only is written on.]

### FLANNEL BED-JACKETS.

"THE HON. MRS. W. H. FREMANTLE," The Deanery, Ripon, writes: In reply to a lady who said she was anxious to have a good pattern of a bed-jacket, as she was intending to provide them for a hospital in her neighbourhood which was shortly to be opened, I have an excellent pattern which I have given to many private friends, and which was made use of for the Ripon Infirmary two years ago, and I would gladly send it. If "H. M. G." will write to me the pattern shall be forwarded immediately.

### PREACHING AND PRACTICE.

"F. T." writes: "Tired Night Nurse" perhaps does not know that in many of our training schools and hospitals at the present time the sisters (day or night) do not have such long hours on duty as the staffs and pros.; they having been through their training and having done a greater amount of work—both mentally and physically—consequently require a little more consideration. Most likely if "Tired Night Nurse" ever gets the position of a sister she will then be of a little different opinion.

[It was not long hours but want of punctuality which was the subject of complaint.—Ed. HOSPITAL.]

### SELECTION OF NURSES FOR PLAGUE IN SOUTH AFRICA.

"AN OLD NURSE" writes:—As one well acquainted with women, men, and manners, I should like to protest against the continual appearance of letters regarding the propriety or impropriety of nurses' conduct. My experience is, that the women who really object to "odd" ways are the ones who say nothing about it in print, but—and here is the point I wish to make—who stop the whole thing at the time. Men are men, but if there is a woman on board ship, or anywhere else, who takes the trouble to show them that "it is not good enough," they behave as gentlemen are supposed to do. They needs must love the highest when they see it. It is the woman with no influence who writes to the papers. *Verb. sap.*

### UNNECESSARY WORK FOR NURSES.

"PROGRESS" writes: I should like to know if it is the rule for nurses trained in the leading London hospitals to have brasses to clean, tanks to polish, forms to scrub, and ward linen to mend. I was trained in a provincial hospital, where this sort of work was unknown to the nurses, and I was much surprised to find on going to a London hospital that they have it all to do. My feeling was that both the nurses and the patients suffered from the arrangement—the former in health from the great strain put upon them, while the latter could not get as much attention as if the nurses were able to devote all their time to nursing them. I cannot see why such work should be expected of nurses, many of whom are gently nurtured women to whom such work is foreign; and it seems to me that ward-maids can do the work much better and cheaper than nurses. Besides, one knows how objectionable a coarse hand is to a patient or for surgical work. Why should a nurse's work be made so much harder than that of many another woman who has to earn her living? I shall be glad to have the opinion of others on this subject.

### AN OPENING AT SIERRA LEONE.

"E. VAN SOMMER" writes from Cuffnells, Wimbledon: I have just returned from spending some months at the Princess Christian Cottage Hospital in Sierra Leone, and desire to bring before your readers the present need of a new sister for this work. The hospital is directly under the Bishop of Sierra Leone, and is for native women and children. There are two wards with about twenty patients, and a very large out-patient department. The staff consists of a medical officer, matron, and two sisters, all English, and four native nurses. The training of these nurses is one of the special objects of the hospital. There is no language to learn as English is spoken and understood. The work is distinctly mission work, and all connected with it must be those who desire to give themselves to Christian as well as to medical work, and must be able to take the services for the out-patients and in the wards, &c. The climate is not healthy, but with care it need not be dangerous. The time of service is one year on the coast and four months' furlough. I have twice spent some months at the hospital as additional worker and member of the home committee, and can give all details to any who wish them.

### SECTARIANISM IN NURSING.

"G. M. W." writes: The statement that the post of matron now vacant in an important Irish hospital is to be filled by a nun again brings forward the vexed question so often raised concerning the position of Roman Catholics in the nursing world of to-day. This step, however retrograde it may appear to lay ideas of modern nursing, is nevertheless the outcome of many difficulties, and under the present condition of things may prove practically to be eminently successful. The question of demand and supply of Roman Catholic nurses of suitable position and experience for these posts will never be satisfactorily answered until the governing bodies of large hospitals and matrons recognise the importance, to say nothing of the justice, of making their staff vacancies as open to Roman Catholics as to members of all Protestant denominations. The present system of training, as well as the uncompromising attitude of matrons towards Roman Catholics, make it almost impossible for members of their faith to obtain the best training and the work most qualified to lead to posts of matrons or superintendents. Why it is hard to understand, especially as no question is asked concerning the extraordinary beliefs or, worst of all, the want of any belief in many of the women who apply for hospital posts to-day. The spirit of unreasoning objection is seen and felt only too keenly by those who have become converts to Roman Catholicism, while those who are Roman Catholics before taking up nursing have little chance at all. Anything but a Roman Catholic is the intolerant cry. A nurse who had entered her hospital as Church of England some years since saw reason to change her views, and rightly took the first opportunity of reporting the fact to her matron. "I hope you have not become a Roman Catholic," said the matron. "No, a Theosophist." "That's all right," said the matron; and this spirit is unfortunately typical, the incident having occurred in one of the most important training schools in London. Why cannot the question of religion be omitted from the schedule to be filled in on entering any training school of importance? The usual references, together with the applicant's personal character and social position, are surely sufficient to testify to her moral fitness for training, and this is the chief point which should exercise the minds of those in whose hands lie the choice and training of women for the most important work of nursing the sick. One's faith is a personal matter: if it influences one's work for good, which, if real, it will do, *tant mieux*; but be the nurse Roman Catholic, Protestant, Mohammedan, or what not, that is a small question, as far as the nursing profession goes, as long as she is not to be found amongst those undesirable women who too often appear in our ranks nowadays, whose habits of smoking, drinking, betting, meeting men in compromising places and at all hours, tinge the nursing profession in such a way that even its best members feel the touch of their shame. Excellent women are to be found in every creed and in every rank, and if more discrimination were shown as to the character of applicants for hospital vacancies, and a less intolerant spirit in the matter of religion, we should not so frequently have to deplore the behaviour of many of our present-day nurses, nor possibly would the committees in Roman Catholic centres find it desirable to appoint nuns to important positions, from whom at least they are sure of work done from the highest motives. No; it rests with matrons in the first place to choose suitable women and then to give them every possible chance of qualifying for the best posts, irrespective of their form of religion. In this way only will the training schools do the work required of them—that is, to supply women for work, which is in itself bounded neither by creed, race, nor caste—and which should be done universally for the benefit of suffering humanity.

### On the Nursing of Convalescents.

It is a recognised fact that many nurses who are skilful and proficient aids in bringing sick folk out of the depths of illness into the desired haven where they would be, are not so successful with convalescents.

Why is this? Because when a patient is really ill the mind becomes comatose, and he is too utterly—mentally and physically—weak to do anything but lie powerless. Even a very restless patient is usually, when seriously ill, somnolent.

But as soon as strength of body, and with it strength of mind, returns, the will-power gradually revives, and the patient is not so blindly acquiescent as before. Then it is that a nurse's tact comes in. Here it may be mentioned that no attempt is made to deal with the treatment of mental cases—always so much more difficult to touch than physical illnesses. A person may be, comparatively speaking, well in body and yet very ill in mind. If a nurse be somewhat indiscriminate, she will find her patient, as he becomes convalescent, more or less difficult to manage. An intelligent convalescent is pretty well able to gauge the character of his nurse, and makes a mental note of any little weakness or strength that she exhibits. People, too, who are at this stage of recovery, are often cantankerous and few exacting. They are keenly sensitive to the slightest thing that grates upon their abnormal nerves. It is not uncommon to hear such a remark as the following: "I don't mind 'X' when I'm well, but when ill I cannot bear her near me!"

Every nurse who has at some time or other been ill herself knows exactly that feeling, and perhaps this is why some of the physically strongest women fail by lack of sympathy towards their patients. Here are a few types of women whom the highly-strung, nervous patient—when well enough to notice things—can scarcely tolerate. The first is the ever-cheerful nurse; the second, the rough and loud-voiced nurse; the third, the too "chatty" nurse; the fourth, the inflexible, hard nurse; the fifth, the uncongenial, almost silent, nurse. These are only some of the many "trying" kinds.

One, whom a convalescent patient once described as "perfect" by reason of her being "talkative when you want to talk, quiet when you wish to be quiet, and who seems to know exactly what you need without being asked for it," is indeed an ideal nurse.

Many private patients on the high road to renewed health find a great trial in possessing a nurse who is not an all-round well-informed woman. A patient, too, will often ask for the newspaper to be read aloud, and therefore it is a great advantage for a nurse to be a good reader, and to have a well-modulated voice—"that excellent thing in woman."

To be self-effacing and sociable enough to join the convalescent in games, &c., does much to lessen the tediousness of recovery. It is just that knowledge of "when and how"—a gift of instinct more than anything else—which tends to make a successful nurse for convalescents. She must be in sympathy with every mood of her patient, and although much gentleness and patience are necessary, yet she should never cease to show her strength of purpose and decision of character. Kindness is not another name for weakness, and the wisdom of the serpent is quite as necessary a quality for a nurse to possess as the harmlessness of the dove.

### Appointments.

BISHOP'S STORTFORD WORKHOUSE INFIRMARY.—Miss E. Wilson has been appointed superintendent nurse. She was trained at St. George's Infirmary, Fulham Road, London.

BRIGHTON THROAT AND EAR HOSPITAL.—Miss Fanny Barrow has been appointed staff nurse. She was trained at Poplar and Stepney Sick Asylum.

GUEST HOSPITAL, DUDLEY.—Miss Ada Kelly has been appointed night superintendent. She was trained at the Devon and Exeter Hospital, where she was subsequently staff nurse, and sister on holiday duty. She was also for some time attached to the private nursing staff. Her last appointment was that of sister of male wards and operating theatre at Ryde Hospital.

HOSPITAL SAMARITANO, SAO PAULO, BRAZIL.—Miss Lucy Jacobs has been appointed matron. She was trained at King's College Hospital, and for the last three years has been on the staff of the Hospital Samaritano.

JESSOP HOSPITAL FOR WOMEN, SHEFFIELD.—Miss Amy E. Robinson has been appointed sister. She was trained at Scarborough Hospital and Dispensary and Glasgow Maternity Hospital, and has since been ward sister in the latter institution.

MAIDSTONE FEVER HOSPITAL.—Miss Annie Brandon has been appointed matron. She was trained at the Preston and County of Lancaster Royal Infirmary.

MEXBOROUGH MONTAGU COTTAGE HOSPITAL.—Miss Janet Cooke has been appointed matron. She was trained at the Guest Hospital, Dudley, and the General Hospital, Nottingham. Subsequently she was theatre sister at the Sheffield Royal Infirmary, night superintendent at Liverpool Stanley Hospital, and assistant matron at the Ilkley Hospital and Convalescent Home.

PLYMOUTH WORKHOUSE.—Miss Dora Joyce has been appointed assistant nurse. She was trained by the Meath Workhouse Nursing Association at the Crumpsall Infirmary Manchester.

ROYAL ISLE OF WIGHT COUNTY INFIRMARY, RYDE.—Miss Annie Gillespie has been appointed night sister. She was trained at the Royal Hospital, Portsmouth, and has since been charge nurse at the Fever Hospital, Tooting.

### Where to Go.

ROYAL ALBERT HALL, Saturday, June 8th, 3.15.—Grand morning concert in aid of the East London Hospital for Children.

ST. JAMES'S THEATRE, Tuesday, July 2nd.—Matinee in aid of the Westminster Hospital at 2.30. Tickets from Mrs. Henry Dickens, 2 Egerton Place, S.W.

### Presentations.

MACCLESFIELD INFIRMARY.—On the evening of May 24th the sisters of the Macclesfield General Infirmary held an "at home," when the nursing staff presented their matron, Miss Noble (upon the occasion of her resignation of her post), with a beautiful travelling bag, with their good wishes and many regrets at her departure. The following evening the servants made her a very nice and useful presentation.

### Echoes from the Outside World.

### AN OPEN LETTER TO A HOSPITAL NURSE.

THE fact that the King on Saturday invited most of the principal members of the New York Chamber of Commerce to Windsor Castle will have caused no surprise to those who remember how often, as Prince of Wales, he has shown marked favours to American men and women. Queen Victoria, though always on the most cordial terms with the American nation, seldom made personal friends with individual members. The party, about twenty-five in number, were first escorted to the Royal Mausoleum at Frogmore and then back through the town to the cloister entrance to St. George's Chapel. Thence the Dean of Windsor conducted them into the Albert Chapel to see the tombs of the Duke of Clarence and the Duke of Albany. Afterwards the King and Queen received their visitors on the East Terrace. The King shook hands with them all, and the Queen and Princess Victoria, who graciously greeted them, were accompanied by three of the children of the Duke of Cornwall and York, the second little boy, Prince Albert, leading his grandmother, or, rather, being led by her. Later on, after the King had conversed in turn with many of the gentlemen, all of whom at his request resumed their hats, he walked about the Terrace and grounds with them and ultimately they adjourned to the Orangery for tea and refreshments, whilst the Royal party partook of theirs on the Terrace. Americans who are well acquainted with the English climate will know that it very seldom allows of *al fresco* meals as early as the first of June.

THERE have naturally been great rejoicings over the birth of a little daughter to the King and Queen of Italy. They were married in 1896, and this is their first child. Some regret is, of course, occasioned that the little stranger is not a boy; but it will be remembered that, though King Edward now sits upon the English throne, the Princess Royal was Queen Victoria's first baby, and that the little King of Spain has two elder sisters. If the baby is at all like its mother it will be extremely pretty, for Queen Helena is one of the most beautiful women in Europe. She is the eldest daughter of the Prince of Montenegro. The little girl has the distinction of being the first member of the reigning dynasty to be born in the Eternal City, and she is to be called Yolanda Margherita. Queen Maria Pia of Portugal, the child's great aunt, and sister to the late King Humbert, is to be the godmother, and the christening is to take place at an early date. English ways and English ideas are to reign paramount at the Quirinal Palace. The nurseries are to be presided over by Mrs. Dickens, who is to act as head nurse, and the King, as well as the Queen, seems desirous to give her full authority to arrange all as she thinks best. In honour of the event a Knighthood has been conferred upon the Premier, and upon some of the other ministers; the King has issued a decree granting an amnesty for Press offences, duels, and crimes committed in the riots of 1898, excepting in the case of persons guilty of homicide; and the Queen has ordered a present in kind and in money to be distributed to the mother of every child born in Rome the same day as the small Princess, the number of recipients of the Royal bounty being thirty-one.

ANOTHER Queen in whom Englishwomen are much interested, the young Queen of Holland, has, with her husband, been paying a visit to the German Emperor at Berlin, and has won golden opinions by her natural grace and simplicity of manner. She was welcomed by the Chief Burgomaster, and the Queen, instead of deputing the duty to Prince Henry of the Netherlands, replied herself in a few well chosen words, saying how much she had always desired to become acquainted with the beautiful City of Berlin. At the banquet in the evening the Emperor William mentioned that the House of Brandenburg-Hohenzollern owed much to the House of Orange, and touchingly commenced his tribute by saying, "Not as strangers are we to greet your Majesty to-day. Once before this house had the honour of being visited by your Majesty, and again we have the pleasure of bidding your Majesty welcome in the same halls. The first time in tender childhood, this time in the full springtide of life by the side of a beloved husband of genuine German stock." In her sweet little reply Queen Wilhelmina expressed the wish of her whole heart that the old and tried relations between the houses might always exist, and in order to emphasise the wish she raised her glass and drank to the health of their Majesties the Emperor and Empress. To an Englishwoman a toast of this kind seems strange, but in Germany, of course, the drinking of healths is regarded as a very important function, and one can imagine how pretty the Dutch Queen looked as she played at being a man. It must have been the more charming because, though so clever, she is such a womanly sovereign.

THE new police order put forth by Sir Edward Bradford will be welcomed by all. Though issued just before Derby Day, so that it might be brought into force at once, it is intended to apply equally to large and jubilant street gatherings. The fashion of squirting so-called scent at passers-by—which much more frequently partakes of the nature of dirty water—is a practice which has only prevailed in later days, but apparently it was so rapidly taken into favour that it has become the terror of decently dressed men and women. Squirters enjoyed themselves vastly on Ladysmith Day, Mafeking Day, and C.I.V. Day, but they must now look back with sadness on past triumphs, for their reign is at an end. A person found using a squirter will be taken before a magistrate, and as a magistrate is not always sitting, the police have authority to detain the offender unless they enter into recognisances for their appearance. As this may not always be practicable, the delinquents may, I suppose, frequently be given a bed in prison for the night. And in order that they may have as little opportunity as possible to offend, persons offering squirts for sale in the streets will be treated as inciters to others to commit assaults, and punished accordingly. I rejoice to think that it is now safe to sing "The Requiem of the Squirt."

I CAME across an interesting summary the other day of an account book kept by a maiden lady who lived in a country town in Staffordshire 170 years ago. A few of the items entered by the thrifty soul are so amusing that I jotted them down for your edification, more especially the little bit which alludes to illness. In 1746 Miss Taylor had a fever, and paid a nurse 12s. for attending her for the period of six weeks! Though the nurse was probably only the old woman of the village, who, being too infirm for anything else, was still considered fit to nurse, the sum of 2s. a week seems an exceedingly modest remuneration; but then money was, of course, worth a great deal more in those times than it is now. The invalid also paid 1s. to a Mr. Moss for bleeding her, but she does not state whether Mr. Moss was the barber or the druggist; and he does not appear to have been the doctor, as elsewhere she makes an entry of £1 1s. paid for "advice in illness." She resided as a paying guest with her nieces and nephews, to whom, as her share of the household expenses, she gave £11 14s. a year, providing her own tea, sugar, and coals. The tea cost her 18s., the sugar 7½d. a pound, but her coals were wondrously cheap, for she got a horse-load for 1s. Her dress materials were, of course, very expensive, blue paduasoy being 11s., and green tabby about 6s. 9d. a yard; but dyeing was less than at present, I fancy, for "dyeing my blue satin nightgown" is tabulated as costing 3s. 3d. Later on she shows that she also possessed a red satin garment for night-wear. These coverings must have looked very gorgeous, especially in the case of a sudden alarm or a fire; but I think a modern nurse would have objected to them somewhat when the fever was on. Perhaps, however, a "sick gown" was provided for such occasions.

### For Reading to the Sick.

THE HEAVENLY WEAVER.

My life is but a weaving
Between my God and me,
I may but choose the colours
He worketh steadily.
Full oft he weaveth sorrows
And I in foolish pride
Forget He sees the upper
And I the under side.

*Anon.*

Thou camest not to thy place by accident.
It is the very place God meant for thee;
And shouldst thou there small scope for action see,
Do not for this give room to discontent.

*R. C. Trench.*

But in truth we live alone as much as we die alone; and we, "whose spirits live in awful singleness, each in his self-formed sphere of light or gloom," need to know and realise that great conviction before we die. We want it in life, to elevate, to consecrate, to purify life; to give it truth and nobleness, to raise it to its real power and hope. This conviction may come indeed at any moment—in the hurry of business, in the hour of joy, in the misery of bereavement, ah! even in the very moment of temptation and the hour of sin, we may learn and feel the startling and essential singleness of the soul.—*R. W. Church.*

My child, thou mayest not measure out thine offering unto me by what others have done or left undone; but be it thine to seek out, even to the last moment of thine earthly life, what is the utmost height of pure devotion to which I have called *thine own self*. Remember that, if thou fall short of this, each time thou utterest in prayer the words, "Hallowed be Thy name, Thy kingdom come," thou dost most fearfully condemn thyself, for is it not a mockery to ask for that thou wilt not seek to promote even unto the uttermost, within the narrow compass of thine own heart and spirit?—*The Divine Master.*

There is no true and constant gentleness without humility; while we are so fond of ourselves, we are easily offended with others. Let us be persuaded that nothing is due to us, and then nothing will disturb us. Let us often think of our own infirmities, and we shall become indulgent towards those of others.—*Fénelon.*

God maketh a great silence, that we may hear distinctly the softest whisper of the still, small voice. And He maketh a great darkness, that we may be able to discern the least and farthest of His stars of truth.—*V. Welby-Gregory.*

This is one result of the attitude into which we are put by humility, by disinterestedness, by purity, by calmness, that we have the opportunity, the disengagement, the silence in which we may watch what is the will of God concerning us.—*Dean Stanley.*

### Notes and Queries.

**The Editor is always willing to answer in this column, without any fee, all reasonable questions, as soon as possible.**

**But the following rules must be carefully observed:—**

**1. Every communication must be accompanied by the name and address of the writer.**

**2. The question must always bear upon nursing, directly or indirectly.**

**If an answer is required by letter a fee of half-a-crown must be enclosed with the note containing the inquiry.**

*Deafness.*

(94) Can you tell me if the treatment offered by the —— Institute would cure my deafness caused by rheumatic fever? Where could I learn lip-reading during my holidays?—*Jessie A. D.*

Have nothing to do with the establishment, it is quackery; (2) Apply, the Director. School for Children and Training College for Teachers of the Deaf, 11 Fitzroy Square, W.; or Miss Isabel Pollock, care of Messrs. Simpkin Marshall and Co., Paternoster Row, E.C., can probably help you.

*Chiropody.*

(95) I wrote in January asking where I could receive instruction in chiropody, but have received no reply—*Sister Anne.*

Your query was answered under the heading "Toilet" on March 30th.

*Drugs.*

(96) Would anyone recommend a home where a trained nurse, wishing to be cured of the habit of taking drugs, would receive maintenance and treatment in return for service.—*X. Y. Z.*

We advise you to advertise.

*Discharge from the Ear.*

(97) Could you recommend the —— Institute as a suitable place at which to receive a course of treatment for chronic discharge from the ear? If not, where is the best place to attend? 2. Could you tell me of a book on diseases of the ear, and their cure?—*A. B. L.*

Certainly not; consult a doctor.

*Biscuits.*

(98) Would anyone kindly tell me if there is any nourishment in Huntly and Palmer's wheatmeal biscuits. I have a patient who fancies them.—*S. W.*

Wheatmeal is more nourishing than white flour. The biscuits are very wholesome and suitable for the sick.

*Male Nurse.*

(99) 1. I am a clerk, 27 years of age, and am anxious to train as nurse in London. What ought I to do?

2. Will my ambulance and science and art certificates help towards my getting into a good school?

3. Would the training which has been confined to a sick asylum, workhouse, or union infirmary, be recognised in the nursing world as thorough?

4. What is meant by "salary will be subject to the Poor Law Officers' Superannuation Act Amendment Act," and "Local Government Board?"—*Ignorant.*

1. Apply to the National Hospital for the Paralysed and Epileptic, Queen's Square, Bloomsbury, W.C. 2. No. 3. Everything depends upon the standing of the training school at which the nurse is trained, as there is no definite standard of training for a male nurse. The hospital named above is the best. The asylums mentioned in the "Nursing Profession: How and Where to Train," are reliable. 4. You can obtain a copy of the "Act" at a law stationer's. The phrase means that deductions are made from the salaries of the employed towards a pension.

*According to Rule.*

(100) There is a lady on the Committee of our District Nursing Association who, since she has become a widow, has resumed her occupation as trained nurse; and who is now in charge of a private case. She has not resigned, nor has she any intention of resigning her position on the Committee, and attends the meetings in uniform. Is this, in your opinion, quite right?—*B. K. G.*

This is a matter which rests entirely between the Committee and the lady in question.

*Medical Library.*

(101) Will you kindly tell me the best place to procure old medical books and also the latest ones. I should be glad of addresses.—*Nurse Frances.*

You might join a good library. The Royal British Nurses' Association, 10 Orchard Street, London, W., or the Trained Nurses' Club, 12 Buckingham Street, Strand, W.C., have libraries for their respective members of standard medicine works useful to nurses. The simplest plan, of course would be to go to a bookseller.

*Free Homes.*

(102) Will you tell me where and how I can get the names and addresses of homes where invalids are received permanently without payment.—*Nurse B.*

You will find a list of homes in Burdett's "Hospitals and Charities," under the heading "Chronic and Incurable."

**Standard Books of Reference.**

"The Nursing Profession: How and Where to Train." 2s. net; post free 2s. 4d.
"Burdett's Official Nursing Directory." 3s. net.; post free, 3s. 4d.
"Burdett's Hospitals and Charities." 5s.
"The Nurses' Dictionary of Medical Terms." 2s.
"Burdett's Series of Nursing Text-Books." 1s. each.
"A Handbook for Nurses." (Illustrated.) 5s.
"Nursing: Its Theory and Practice." New Edition. 3s. 6d.
"Helps in Sickness and to Health." Fifteenth Thousand. 5s.
"The Physiological Feeding of Infants." 1s.
"The Physiological Nursery Chart." 1s.; post free, 1s. 3d.
"Hospital Expenditure: The Commissariat." 2s. 6d.

All these are published by THE SCIENTIFIC PRESS, Ltd., and may be obtained through any bookseller or direct from the publishers, 28 and 29 Southampton Street, London, W.C.

### Travel Notes.

By Our Travelling Correspondent.

### LXXIII.—HOW TO SEE THE GLASGOW EXHIBITION.

In spite of the most strenuous determination to abide by the time-honoured advice to cut one's garment according to one's cloth, a visit to Scotland or Ireland always means somewhat extensive outlay. It is hard that travelling in our own country should be beyond the means of many of us, and that not a few experienced continental tourists are obliged to confess to but little knowledge of the beauties of the British Isles. This is largely due to hotel and lodging-house keepers, who are in haste to be rich, and who ask prices for their accommodation that are prohibitive to those of slender means; but in justice it must be admitted that the fault does not wholly lie with them. The travelling Briton in his own country is often exacting, and demands a supply of creature comforts that he would cheerfully forego abroad. For instance—very few Englishmen are content at home to breakfast on coffee and rolls; they demand eggs, rashers, and perchance a succulent cutlet, and these delicacies cost money. He has to pay at least 2s. for his breakfast, and it is only in modest hostelries that he will be let off at that price; whereas in France or Germany his simple meal will cost him 10d. Thus, exaction from the traveller and a not unnatural desire on the part of the host to make his harvest during a short season, keeps up the prices, and the steady flow of tourists across the Channel continues.

### Means of Reaching Glasgow.

A third-class tourist return from London is £2 12s., being a saving of 11s. on the ordinary fare. From Liverpool it is only £1 7s. There are also some cheap short excursions for three and eight days, for the benefit of sightseers to the Exhibition, which I can tell you of, should you intend going. That from London for eight days is only £1 6s. But unless impossible to extend your holiday, it is hardly worth while taking so long a journey without seeing some of the wonderful beauties of the scenery surrounding Glasgow.

### Accommodation in the City.

You cannot reckon upon anything under 7s. 6d. or 8s. per day for a short stay, and even for those terms it is necessary to look about. Men who can go anywhere, and don't mind roughing it, can find accommodation at lower rates, but not so women, and outside the big towns prices are even less moderate, on account of lack of competition. Anyone making the trip had better write to me saying exactly the amount of time and money at their disposal and I will advise to the best of my ability.

### The Exhibition Itself.

There is naturally an immense amount to be seen, and it is therefore well to arrange in your own mind some kind of plan of sightseeing, so that no time may be wasted. If you intend to devote three days to the show (entrance 1s., children 6d.) settle what sections you will see and what may be avoided, for when I remind you that the buildings cover 100 acres, you will understand that much must be left unseen, and that is no loss when tastes differ so much. I love machinery, but others hate it and abhor the noise and smell. I loathe everything to do with China and Japan, and it gave me no pain to forego looking at the really wonderful things in the Japan section; but then there are those who love such things and marvel again at my devotion to the "Hall of Clans."

Undoubtedly one of the greatest attractions is the Permanent Art Gallery, which will endure when the Exhibition of 1901 will have disappeared. The "Hall of Clans" is to me (and all who can claim some connection with the Highlands) a most enthralling spot.

The Scottish Home Industries Association attracts large crowds; the section is always full of spectators gazing at the picturesque spinning wheels in motion preparing the wool for the homespuns and tweeds in which we all delight for our tailor-made costumes.

### The Tea-Rooms.

The success of these is enormous; they are thronged all the afternoon; wearied sightseers rest and are thankful, imbibing, with the fragrant cup, fresh energy to attack new fields of exploration. What a change from the horrors of the 1851 Exhibition, the weariness of which our parents relate to us with sad reminiscence. Afternoon tea was then an unknown luxury; nothing came to break the dead monotony of the lagging hours after luncheon, when perhaps some piquant dainty had created a raging thirst; from section to section stern duty dragged reluctant and weary legs till human nature could bear no more, and the sufferer gladly left the scene of torture, cross, tired, and often with a racking headache. The tea-rooms are numerous, but there are four, especially charming, and yet very unpretending, called The Bell, The Ring, The Bird, and the Tree; these emblems occur in the Glasgow arms.

In this exhibition there seems a merciful absence of the abominable "side shows," tawdry and trumpery, that so often mar these monster undertakings.

### Glasgow Itself.

I have left myself but little space to speak of the great town of St. Mungo. There is an excellent little guide book published by Pearson, Lim., at the modest price of 1s., which will give the Exhibition tourist all necessary information about the show itself, the town, and the surrounding neighbourhood, with accounts of the best way of reaching spots of interest which abound all round. I hope next week to tell you something of inexpensive excursions which may be made by those having a week or ten days in Scotland.

The Cathedral of St. Mungo is naturally the first thing to be seen in Glasgow. The splendid crypt, which is called the *Laigh* or "low kirk," has forty windows, and the roof and superstructure is supported by more than sixty vast clustered columns. The ancient Tolbooth exists no longer, the only remaining fragment is the cross steeple, but there are still a few ancient streets, the Briggate, Saltmarket, Gallowgate, and High Street which retain something of a seventeenth-century flavour; but taken as a whole Glasgow is depressingly modern.

### TRAVEL NOTES AND QUERIES.

St. Andrews, Edinburgh, and Glasgow (Scotland).—Your cheapest way to and from London is by sea thus—From London to Dundee every Wednesday and Saturday from Wapping, 1st class £1 2s. 6d., 2nd 15s. Dundee is a little north of St. Andrews, but very near. Dundee to Glasgow *viâ* Edinburgh will be about 8s. by train. From Glasgow to London four times a week; 1st class £1 10s., 2nd is not quoted, but will be about 17s. Second class is not luxurious, but I have made inquiries and find it is quite endurable. You will pay for your food as you have it, and if you go 1st you must give the stewardess 2s. 6d., if 2nd 1s. From Seaton to London you cannot take a return ticket because of Maidstone, and must pay the ordinary price. You cannot get a Cook's or Gaze's right through, but there is no difficulty between Seaton and London. Neither agent has anything that will do for you, but Messrs. Gaze and Sons, who do most of my work, will get your steamer tickets for you if you write to them, 150 Piccadilly, London. Hotels in Edinburgh—the "Old Waverley" and the "Maitland." These are temperance hotels, and more reasonable. Ask for a room on the third floor, and you may keep your expenses down to 8s. 6d. per day. I should write first and inquire terms. Scotch hotels are dear. In Glasgow try "Cranston's Waverley" or "Drummond's Temperance." "Duncan's Temperance," Union Street, professes to board you for 7s. per day. Write to them and ask. Let me know if I can help you further.

Davos Platz in the Autumn (Anxiety).—The cheapest way is by Southampton and Havre, 2nd class, £3 18s. 6d. There are several pensions that will take you from 5 francs; if you can manage that I will give you their names, but space is precious. If a party of several for a long stay I think you might even make more advantageous terms. Some people think Dâvos delightful in summer, and, being so high, I should think it must be healthy, but this is a question for your doctor to decide. I am afraid there is very little chance for you to help payments in the way you suggest. Possibly when there you might get repairing and altering to do, and the nurse *perhaps* some cases, but I think it very doubtful and don't want you to be too hopeful. If she has to go alone I fear there is no place where she could be, as you say, "looked after." Once there possibly the Chaplain might suggest something to you if she must remain alone. It is a gay little place, and people are very kind to each other. If the patient's case is so hopeful I should recommend her studying foreign languages and qualifying herself in some line of life, as a lady's maid, waitress, or something of that kind, to remain abroad always in the spring and winter, so as not to encounter our changeable climate. The calling of a governess is not exhilarating or healthy—personally I would much rather be in a shop or take a post as a trusted upper servant. If I can help further let me know, and when you have thought it over, if you find it can be managed, I will send you addresses.

## Figures and Tables

**Figure f1:**
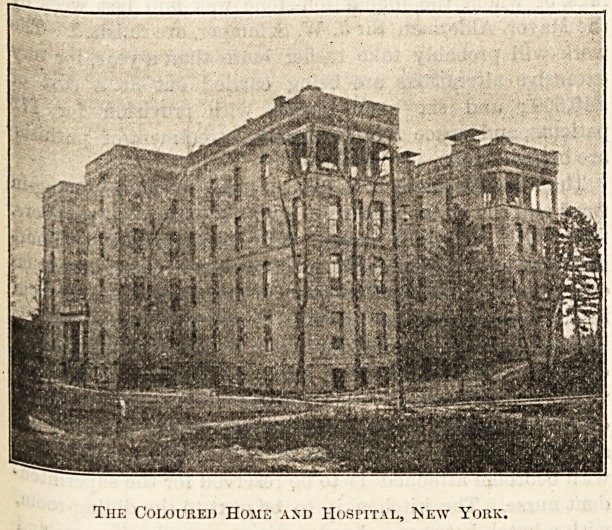


**Figure f2:**
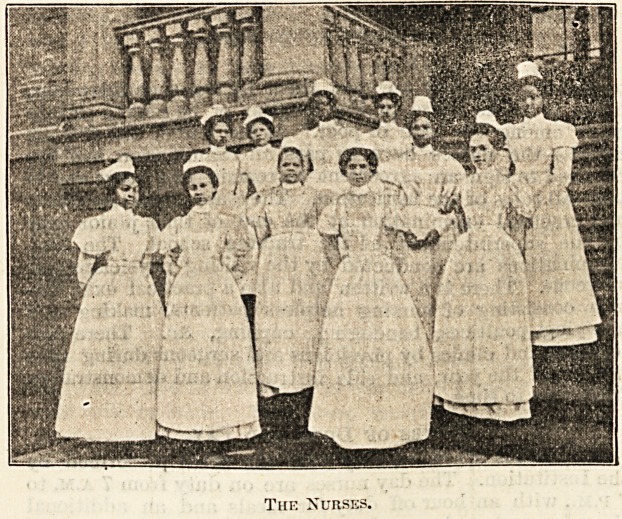


**Figure f3:**